# Tribological Aspects of Sheet Titanium Forming

**DOI:** 10.3390/ma16062224

**Published:** 2023-03-10

**Authors:** Wojciech Więckowski, Janina Adamus, Marcin Dyner, Maciej Motyka

**Affiliations:** 1 Department of Technology and Automation, Faculty of Mechanical Engineering and Computer Science, Czestochowa University of Technology, 69 Dąbrowskiego St., 42-201 Częstochowa, Poland; 2 Department of Civil Engineering, Faculty of Civil Engineering, Czestochowa University of Technology, 69 Dąbrowskiego St., 42-201 Częstochowa, Poland; 3 Department of Advanced Computational Methods, Faculty of Science and Technology, Jan Dlugosz University in Czestochowa, 13/15 Armii Krajowej Ave., 42-200 Częstochowa, Poland; 4 Department of Materials Science, Faculty of Mechanical Engineering and Aeronautics, Rzeszow University of Technology, 12 Powstańców Warszawy Ave., 35-959 Rzeszów, Poland

**Keywords:** sheet metal forming, ecological lubricant, boric acid, titanium sheet

## Abstract

Friction, wear, and lubrication are inherent to all metal-forming processes. Unfortunately, they are particularly troublesome when forming titanium materials, which tend to form titanium buildup on the working surfaces of the forming tools. Lubrication is one of the most effective ways to separate contacting surfaces and eliminate galling, thus reducing tool wear. The paper presents the tribological aspects of titanium sheets forming using environmentally friendly lubricants with the addition of boric acid. The lubricant’s effectiveness was assessed on the basis of technological tests, such as the strip drawing test, the Erichsen cupping test, and the formation of spherical drawn parts in industrial conditions. Moreover, the results of the numerical simulation of forming a titanium hat-shaped part are presented. Numerical calculations of forming processes were performed using the PamStamp 2G system based on the finite element method. Both experiments and numerical analyses showed the positive effect of lubricants with boric acid on sheet titanium forming.

## 1. Introduction

The popularity of sheet metal forming processes results from the possibility of manufacturing light and durable products, both small and large ones, with simple or complex geometry, e.g., parts both for the transport and construction industries, as well as for household goods and small metal accessories. Sheet metal forming enables the production of almost ready-made, drawn parts without the need for machining. The leading role in sheet metal forming is played by the automotive industry, the development of which is closely related to the development of the steel industry. However, the growing expectations of consumers force the use of new materials and technological solutions. The range of materials used in the sheet metal forming industry is expanding every year. Demands for increased corrosion resistance led to the use of galvanized sheets. Current activities are aimed at reducing the weight of the structure. Attempts are being made to introduce high-strength alloy steels, such as aluminum or titanium, and their alloys, including welded blanks [1,2,3]. Unfortunately, the use of these materials is associated with solving many technological problems, including the spring back and low formability of sheets [4], or the tendency to create a buildup of the formed material on the tools [5,6,7]. Particularly noteworthy are titanium materials, which, up to a temperature of 600 °C, have the highest specific strength (the ratio of mechanical strength to density) among all metal construction materials [8]. What is more, they are resistant to most corrosive environments. Therefore, titanium sheets are of great interest in the biomedical and aerospace industries [9,10,11,12].

In order to increase the formability of titanium sheets and reduce spring back, forming at elevated temperatures is used [13,14,15], including superplastic forming [16,17]. The influence of temperature, coating, and lubrication on tribological characteristics in hot forming is discussed in detail in [18]. Quite a lot of work is on hot incremental formation [19,20,21,22]. Generally, incremental forming, especially cold incremental forming, was developed as an alternative die-less sheet forming process, but it is effective only in prototyping drawn parts and small batch manufacturing. Additionally, product surface quality poses a big problem due to the surface roughness [23] and traces left by the forming tool on the drawn part surface. In order to improve the surface quality of products, lubricants such as a mixture of WS2 powder and oil [20] or self-lubricating coatings [24] are used. Guoqiang Fan et al. [19] see the solution to this problem in the use of an appropriate lubricant. According to the authors, a lubricant film of nickel matrix with MoS_2_ allows for obtaining workpieces with high surface quality.

Many authors, including Wang and Yang [25] and Fratini [26], emphasize that friction is a key factor affecting the value of the drawing force, uniformity of metal flow, life of the tools, and thus product quality. Although much effort has been made to study the mechanism of friction, due to the fact that it is a highly non-linear physical phenomenon that depends on many factors, various issues still need to be clarified and investigated. Particularly dangerous is the phenomenon of the formation of a metallic buildup of deformed sheet metal on the working surfaces of the tools. During sheet metal forming, the drawn part’s surface increases even several times compared to the blank surface. Then, there is direct contact of the sheet material not covered with oxides with the working surfaces on the tools. The large proximity of the contacting surfaces and low relative speeds favor the occurrence of adhesive wear on the tools. Initially, the deformed material slides over the tool surfaces, resulting in microscopic scratches on the sheet surface and local transfer of the deformed material to the tool working surfaces as a result of adhesion. Then, there is an accumulation and growth of the buildup, followed by chipping, which initiates abrasive wear. Further sliding of the contacting surfaces leads to the re-transfer of the deformed material to the tool surfaces and the repetition of the cycle of creating and shearing adhesive joints. The increasing amount of microdamage results in damage to large areas of the tool surfaces, and consequently also the drawn parts surface. This wear mechanism occurs in the event of insufficient lubrication or when the lubricant film is broken due to high surface pressures of up to 100 MPa [27]. The buildup of the formed material on the tools is a technological problem associated with titanium sheet forming that is difficult to control [12,28].

At the beginning of the 21st century, research on new technologies that would make it possible to reduce tool wear caused by friction in plastic forming processes was intensified [5,6,29,30,31]. Kataoka et al. (2004) proposed the use of ceramic dies, while Harada et al. proposed the use of plastic dies. Peter Frohn-Sörensen et al. [32] recommended coating the tool working surfaces with titanium nitride (TiN) using plasma-assisted chemical vapor deposition (PACVD). Mori et al. [5], with regard to forming titanium sheet products, which are increasingly used in industry and medicine, suggested the use of sheets commercially colored by the electrochemical method, thanks to which an oxide layer is produced that effectively prevents galling in multi-stage cold deep drawing. Adamus et al. [33] advised to cover the working tool surfaces (dies and blank-holders) with anti-adhesive coatings, providing additional protection against the formation of protrusions of the deformed material at the time of breaking the lubricant film due to the high normal pressure occurring in the sheet metal forming processes. In their opinion, chromium, aluminum bronze, and Cr/CrN-(a-C:H) multilayers seem to be effective in forming titanium sheets. A completely different approach to this issue is discussed in [34]. The authors propose a novel tribological system that uses volatile media, such as liquid carbon dioxide and gaseous nitrogen, introduced directly into the friction zones between the tool and the sheet material during deep drawing under high pressure through special laser-drilled microholes.

However, technological oil lubricants are most often used to reduce the negative effects of friction. Unfortunately, they have a negative impact on the environment. The growing awareness of the degrading influence of human activity on the environment means that all manufacturing processes must be reassessed not only in terms of their technological potential but also in terms of their impact on humans and the environment. This also refers to the sheet metal forming processes, which are costly in terms of material and energy consumption [35,36]. Therefore, even small unit savings bring huge profits in the case of mass or high-volume production. Taking into account the tribological aspects of sheet metal forming, it is important to reduce lubricant waste, which often contains environmentally harmful additives such as chlorine, phosphorus, or sulfur [37]. The action of these additives, known as extreme pressure (EP) and antiwear (AW) additives, is based on a thermochemical reaction with a frictional pair surface under high temperature and pressure to form a low-shear strength boundary lubricating film. Li et al. [38] proposed to substitute traditional EP and AW additives with nanoparticle EP and AW ones, whose tribological performance is mainly based on mending and polishing as well as rolling bearing effects.

In sheet metal forming, apart from lubricants based on mineral and synthetic oils, water-based lubricants are also used, although somewhat less frequently. Chen et al. [39] emphasized that water can be an ecological lubricant base, provided that the lubricant additives, apart from being friendly to humans and nature, are also properly prepared. Solid PVC (polyvinyl chloride) and polyurethane films, molybdenum disulfide, and graphite [40] lubricants are also used. Proper lubrication is especially important when forming titanium sheets, as they are very susceptible to galling [5]. According to the authors, using the bronze dies or calcium stearate lubricant, a long commercial pure titanium cup having a height-to-diameter ratio of six can be successfully formed without intermediate annealing.

The technique of minimum quantity lubrication (MQL), based on the concept of near-dry machining, is currently being explored as an alternative to conventional lubrication [41]. This is very important because during sheet metal forming, the lubricant operates in an open loop, i.e., it is “picked up” by the deformed material and remains on the drawn-part surfaces until it is washed off before coating. Thus, the lubricant is irretrievably lost in subsequent forming operations. In order to minimize hazardous lubricant waste, some authors propose to replace technological lubricants based on mineral oils with green lubricants [28,42], e.g., based on vegetable oils, which are not only biodegradable substances but also, due to the presence of long fatty acid chains, have a good ability to adhere to metal surfaces, thus ensuring good lubrication in boundary friction conditions [43,44]. Deshmukh et al. [45] and Chowdary et al. [46], however, point out that vegetable oils degrade at elevated temperatures, which limits their use to cold-forming processes. Additionally, lubricants based on vegetable oils are subject to aging and bacterial spoilage. In order to improve the lubricating properties, various additives are added to base oils, such as graphite [40], MoS_2_ [47], h-BN [48], or boric acid [49]. The layered structure of these materials ensures low friction resistance. Most of these materials have superlubricating properties, which means that the coefficient of friction (*CoF*) is less than 0.01 [50]. Farfan-Cabrera et al. [42] proposed microalgae lubricants as potential green substitutes for mineral and synthetic oils, as well as vegetable ones.

Studies on frictional resistance in sheet metal forming processes are not limited to experimental tests performed on the special tribotesters with the use of advanced measurement techniques, such as a scanning electron microscope, X-ray photoelectron spectroscopy, or energy dispersive spectroscopy (EDS), used to identify the morphology and chemical composition of the contacting surfaces before and after tests. Works in the field of numerical modeling of sheet metal forming processes, taking into account the effect of friction on the material flow [51,52], including the use of multi-layer artificial neural networks (ANNs) and backward elimination regression for the prediction of values of the *CoF* [53], are also carried out. A special role in numerical analysis is played by forming limit diagrams (FLDs), which make it possible to predict the behavior of the sheet during forming [54,55].

Joining the search for pro-ecological solutions aimed at sheet metal forming processes, the authors of the work present the tribological aspects of forming titanium sheets using environmentally friendly lubricants with the addition of boric acid. The lubricant’s effectiveness was assessed on the basis of technological tests, such as the strip drawing test, the Erichsen cupping test, and the formation of spherical drawn parts in industrial conditions. In addition, the authors present the results of numerical simulations of forming a titanium hat-shaped part. The numerical calculations of forming processes were performed using the PamStamp 2G system based on the finite element method.

## 2. Goal, Scope, Materials and Methods

The main goal is to develop environmentally friendly technological lubricants for forming titanium sheets that would reduce the forming resistance at least to the same extent, as in the case of commonly used lubricants based on mineral oils. Boric acid (H_3_BO_3_) was selected as the main lubricating remedy because its layered crystalline structure, such as the structure of graphite, MoS_2_, or h-BN, allows for a significant reduction in the *CoF*. In addition, boric acid, unlike graphite and MoS_2_, does not cause hard-to-remove dirt on the surface of products. What is important is that it is cheap and harmless to the environment. Due to the low solubility of boric acid both in water and oils, the following methods were used to apply it to the sheet surface:

- No 1: Boric acid crushed in a high-speed mill was sprayed on a thin layer of rapeseed oil previously applied to the titanium sheet just before the forming operation [56]. The use of rapeseed oil was dictated by its availability in the local market and low cost of production;
- No 2: A 25% boric acid solution in methyl alcohol was sprayed on the titanium sheet.

For comparison, the tests were also performed for the sheet covered with rapeseed oil alone—No 3; In the condition of technically dry friction—No 4.

**Figure 1:**
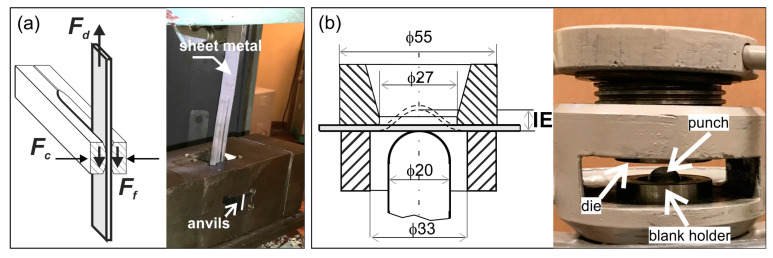
Experimental tests: (**a**) the strip drawing test (*F_d_*—drawing force, *F_c_*—clamping force, and *F_f_*—frictional force); (**b**) the Erichsen cupping test.

The effectiveness of these methods was assessed in two laboratory tests, the schemes of which are shown in Figure 1:

1. The so-called strip drawing test, which was described in detail in [57] (Figure 1a);
2. The Erichsen cupping test [58] (Figure 1b).

**Figure 2:**
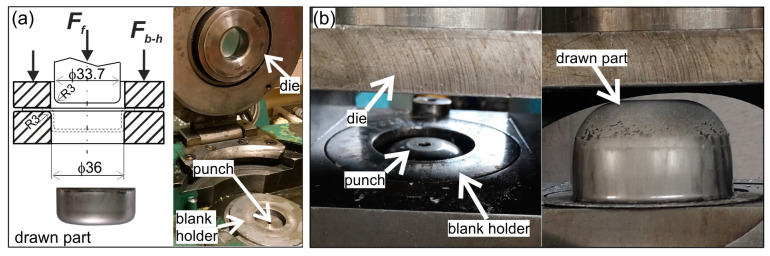
Forming of: (**a**) cylindrical drawn part (*F_f_*—forming force, *F_b-h_*—blank holder force); (**b**) hat-shaped part.

Moreover, in industrial conditions, the cylindrical drawn parts (Figure 2a) were formed on the double-action deep drawing hydraulic press, and the hat-shaped parts (Figure 2b) were formed on the hydraulic press having a spring-pressed blank holder.

The following sheets were tested: commercially pure titanium Grade 2 sheet having a thickness of 0.85 mm and titanium alloy Grade 5 (Ti-6Al-4V) having a thickness of 1.0 mm.

The chemical composition of the sheet materials and their mechanical properties are given in Table 1 and Table 2, respectively. The basic mechanical parameters were determined in a static uniaxial tensile test in accordance with [59].

During the strip drawing test, 20-mm-wide titanium sheet strips were drawn between two counter samples (anvils) made of NC10 tool steel for cold work according to Polish standard PN-86/H85023. The chemical composition of this steel is: C–1.5 ÷ 1.8, Si–0.15 ÷ 0.4, Mn–0.15 ÷ 0.45, P–max. 0.03, S–max 0.03, Cr 11 ÷ 13 (in wt.%). The hardness of the counter-samples was 60 HRC. The working surfaces of some counter-samples were prepared by grinding (Ra ≈ 0.28 µm, Rz ≈ 2.2 µm) and the other by polishing (Ra ≈ 0.02 µm, Rz ≈ 0.1 µm). The strips were cut out of the sheets in a direction perpendicular to the rolling direction. The surface roughness of samples (strips) is summarized in Table 3. The roughness and topography of the working surfaces of the anvils were measured using the TALYSURF 120 contact profilometer (Taylor Hobson). The roughness measurement was carried out on the measuring section l_n_ = 4 mm, with the elementary section l_r_ = 0.8 mm. The surface topography was measured on the working surface of an anvil with dimensions 9 × 20 mm and a step of 0.02 mm. The working surfaces of the anvils were prepared using the AutoMet 250 Buehler grinder and polisher. The dry grinding of the surfaces was carried out with 200 Grit sandpaper. Wet polishing was carried out using 2000 Grit sandpaper, polishing cloth, and diamond suspension Poly-Top-Duo 3 μm.

The sheet titanium strips were drawn with stepwise increasing pressure from the anvils on the sheet. The strip drawing test was carried out 3 times for each frictional condition (with and without lubricant), and the results were averaged.

In the strip drawing test, the lubricant’s effectiveness was assessed on the basis of its coefficient of friction, which was calculated from the following formula:

*CoF* = *F_d_*/2*F_c_* (1)

where *F_d_*—drawing force, *F_c_*—clamping force; both forces were measured during the tests (see Figure 1a).

Typically, the Erichsen cupping test is used to determine the stretch-forming capacity of sheet metals. The sheet clamped between the blank holder and the die is formed with a rigid ball punch until a continuous crack appears in the sheet. The cup height at cracking moment, marked as Erichsen index IE in mm, informs about the ability of the sheets into plastic deformation. In this work, the Erichsen cupping test was used to evaluate the lubricant’s effectiveness. Both cup height at the moment of cracking as well as the location and path of the crack attest to the frictional resistance between the deformed sheet and tool surfaces. The spherical punch had a diameter of 20 mm, while the sheet blank was square in shape with dimensions of 65 × 65 mm.

The other forming tests were carried out in a forming plant. The tools for forming the cylindrical parts and the hat-shaped ones consisted of the die, blank holder, and punch. The blank holder forces, both for the cylindrical and hat-shaped parts, were selected so that the sheet blanks could slide out from under the blank holders but without causing wrinkling of the sheets. Thus, in the case of cylindrical cups, the bottom part was formed by stretching, and the cylindrical wall was formed mainly by drawing. The cylindrical cups were formed from a sheet disc with a diameter of 55 mm using the cylindrical punch with a diameter of 33.7 mm and the die and the blank holder with an inner diameter of 36 mm. The effectiveness of lubricants, which were applied to the blank holder and die, was assessed by the value of the forming force.

The hat-shaped parts were used to verify the results of the numerical simulations of the sheet titanium forming process. Numerical simulations were carried out with the PamStamp 2G program, based on the finite element method. In the numerical simulations, the influence of friction on the material flow, strain, and stress distribution was analyzed. The actual hat-shaped drawn parts were formed from the disk blank with a diameter of 145 mm using the cylindrical punch with a diameter of 77.5 mm, the die with an inner diameter of 80.0 mm, and the blank holder with an inner diameter of 84.0 mm. The spherical, hat-shaped cups were formed both by stretching and drawing. After the forming process, the cups were cut into two equal parts along the plane of symmetry of the drawn part. The measured wall thicknesses on the drawn part sections were compared with the values calculated as a result of numerical simulations.

## 3. Results and Discussion

### 3.1. Strip Drawing Test

**Figure 3:**
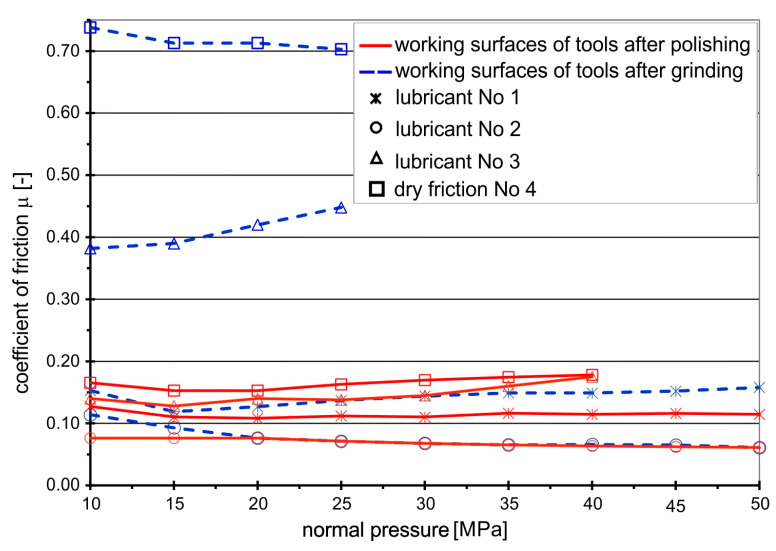
Coefficients of friction for frictional pair “titanium Grade 2–steel NC10”.

**Figure 4:**
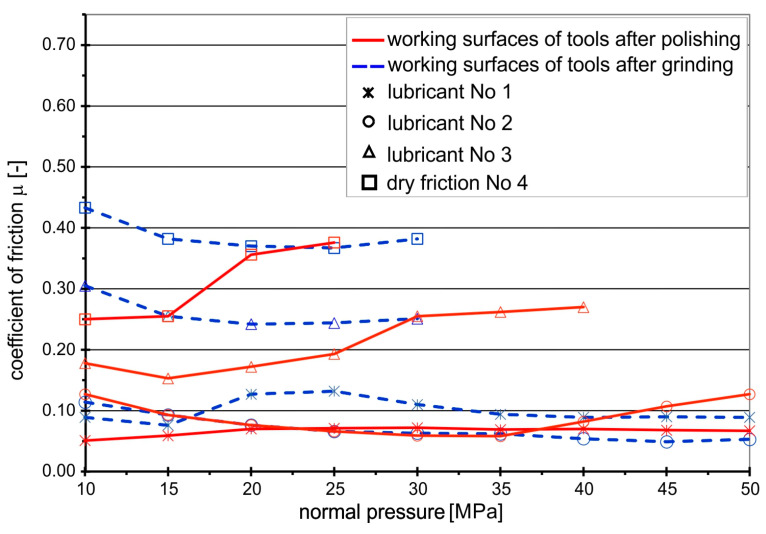
Coefficients of friction for frictional pair “titanium Grade 5–steel NC10”.

The coefficients of friction for the analyzed frictional pairs “titanium–steel” under the conditions of technically dry friction and in the presence of the tested lubricants, determined in the strip drawing test, are shown in Figure 3 and Figure 4. The coefficients of friction for the frictional pair “titanium Grade 2–steel NC10” are presented in Figure 3.

In the case of this frictional pair, in the absence of lubrication, a clear influence of the machining method (grinding, polishing) of the anvil working surfaces on the values of the coefficient of friction is observed. Drawing the Grade 2 titanium strips between the ground anvils results in near-instantaneous galling with a *CoF* of 0.7. Polishing the surface reduces the coefficient of friction by more than 3 times, to just under 0.2. In this case, the first marks of galling appear at the normal pressure of 20 MPa, and then the friction coefficient increases with the increasing amount of titanium buildup to *CoF* = 0.18. Complete seizing occurred at a normal pressure of 39 MPa.

When drawing Grade 2 strips between ground anvils in the presence of rapeseed oil, the coefficient of friction decreases to the value of *CoF* = 0.38, but rapeseed oil effectiveness decreases at normal pressures above 15 MPa, and the *CoF* begins to grow because the oil layer brakes and the first marks of galling appear. Only the addition of boric acid improves the durability of the lubricating film and significantly reduces the coefficient of friction.

In the case of polished surfaces, rapeseed oil slightly reduces the coefficient of friction compared to non-lubricated surfaces but is completely ineffective after reaching a normal pressure of 39 MPa. As in the case of ground surfaces, the addition of boric acid effectively reduces the coefficient of friction and extends the durability of the lubricating film. The lowest values of the coefficient of friction occur when they are lubricated with a solution of boric acid in methyl alcohol, regardless of the surface finish of the anvils, i.e., whether they are ground or polished, the *CoF* value was at a level of 0.05. The use of boric acid sprayed on rapeseed oil and boric acid dissolved in methyl alcohol ensured the maintenance of the lubricating layer in the entire range of the tested pressures, which effectively protected the contacting surfaces from seizing.

The values of the friction coefficients were slightly different when drawing the strips of the Grade 5 titanium sheet, which is characterized by higher mechanical parameters and much lower drawability than the Grade 2 sheet (Figure 4).

As in the case of drawing the Grade 2 titanium sheet, the highest *CoF* values occurred under conditions of technically dry friction on the ground surfaces of the anvils. However, the *CoF* values were lower (*CoF* = 0.47 ÷ 0.40) compared to the frictional pair “Grade 2-steel NC10” (*CoF* = 0.7). After the initial decrease in the *CoF* value and a slight stabilization at the level of 0.37 at the pressure of 15–25 MPa, further increasing the pressure to 29 MPa causes a rapid growth of titanium buildup on the surface of steel anvils, leading to seizing.

Polishing the working surfaces of the anvils reduced the coefficient of friction to 0.25, but only at small normal pressures up to 15 MPa. Further increasing the pressure of the anvils on the drawn sheet strips resulted in increased adhesion of titanium to the steel anvils and, consequently, seizing. The introduction of rapeseed oil between the contacting surfaces of the metal sheet and the tool, both for ground and polished surfaces, leads to a decrease in the *CoF* of about 20 ÷ 30% for the ground anvils and about 50% for the polished ones, but only up to a certain point. After reaching the normal pressure of 29 MPa, the *CoF* value reaches 0.25, after which a further increase in pressure causes the oil film to break and the process of sticking titanium particles to the tools begins. Only the addition of boric acid increases the durability of the lubricating film and reduces the *CoF* to a level of 0.07 ÷ 0.09, with lower values noted for the polished tool. The lowest values of the coefficient of friction at a level of 0.05 occur when a ground tool is lubricated with boric acid dissolved in methyl alcohol. For polished tools, with the increase in normal pressure above 34 MPa, an increase in *CoF* from 0.06 to 0.13 at a normal pressure of 49 MPa was observed, which proves that the lubricating layer is broken and adhesive wear starts.

The main reason for the higher *CoF*s at the beginning of the tests is the transition from static friction (higher *CoF*) between the sample and anvils to kinetic friction (lower *CoF*). In addition, at the beginning, there are higher frictional resistances associated with the higher viscosity of the oil. As the test continues, frictional heat is generated, which reduces the viscosity of the oil, and thus the frictional resistances also decrease.

**Figure 5:**
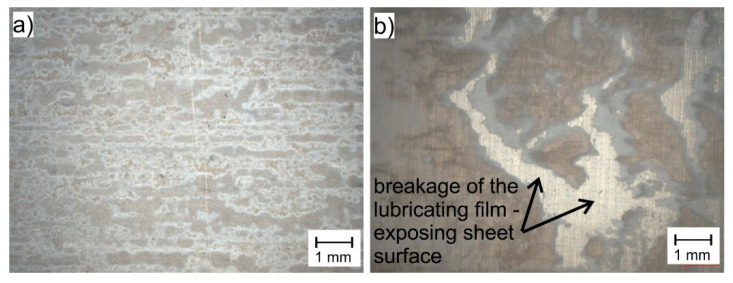
View of sheet surface with layer of lubricant No 2 sprayed on: (**a**) Grade 2 titanium sheet; (**b**) Grade 5 titanium sheet after strip drawing test.

The analysis of the graphs presented in Figure 3 and Figure 4 shows that the effectiveness of lubrication is manifested not only by reducing the frictional resistance (lower coefficient of friction), but above all by increasing the durability of the lubricating film, which is particularly important in the case of sheet metal forming processes, where high normal pressures occur. Even if the presence of lubricant only slightly reduces friction, lubrication is still necessary because it prevents the formed materials from sticking to the working surfaces of the tools, which in turn leads to degradation of the surface quality of the drawn parts. The use of a lubricant in the form of boric acid as an additive to rapeseed oil effectively protects the frictional surfaces against direct contact, both in the case of Grade 2 and Grade 5 sheets. Lubricant in the form of boric acid dissolved in methyl alcohol effectively separated the rubbing surfaces in the case of the Grade 2 sheet (Figure 5a—homogeneous lubricant layer), while in the case of drawing the Grade 5 sheet, the breaking of the lubricating layer was observed at increased normal pressure (Figure 5b—breakage of the lubricating films), which led to an increase in the *CoF* value.

### 3.2. Erichsen Cupping Test

**Figure 6:**
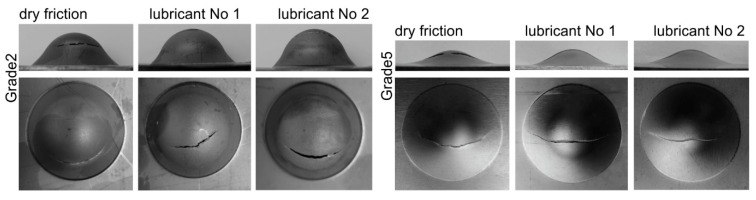
View of cups after the Erichsen cupping test.

The results of the Erichsen cupping test are presented in Figure 6 and in Table 4. The tests were carried out without lubrication and in the presence of lubricants containing boric acid (No. 1 and No. 2), i.e., those that effectively separated the rubbing surfaces during the strip drawing test and ensured the lowest frictional resistance.

Friction occurring between the deformed material and the working surface of the tool plays a major role in the sheet metal forming process. It affects the distribution of strains and limits the height of the drawn part that can be obtained without cracking. During the Erichsen cupping test, the sheet thins as a result of the biaxial stretching. Deeper cups are achievable due to a more even distribution of stresses/strains in the sheet material. The friction between the sheet and the punch prevents the material from flowing away from the pole of the drawn part, and thus the thinning of the sheet occurs outside the area of contact between the sheet and the punch, hence the crack starts at a certain distance from the drawn part pole and runs in the circumferential direction. The greater the share of friction forces between the formed sheet and the punch, the further from the pole the narrowing is formed and, consequently, the crack. In the absence or reduction of frictional resistance, the greatest thinning of the sheet occurs in the central part of the cup, and the crack appears near its pole.

The difference in the failure mode in the case of forming Grade 2 and Grade 5 titanium sheets under dry friction is due to the drawability of these materials (a Grade 2 titanium sheet has much higher drawability than a Grade 5 one), which means that the contact surface and frictional forces between the tool and the sheet are different, so the strain distribution on the sheet surface is also different, and thus the location and course of the crack are different. The greater drawability of the Grade 2 sheet means a larger contact zone of this sheet with the surface of the tool, and because the thinning of the sheet occurs beyond the contact zone of the tool with the sheet, the cracking of the Grade 2 sheet appears further from the drawn part’s pole.

For each sheet, the use of lubricant with boric acid resulted in an increase in the drawn part height at the time of crack initiation. The location of the crack indicates a reduction in friction. Lubricant No. 1 is preferred for forming Grade 5 sheets. Forming results in the presence of boric acid dissolved in methyl alcohol were only slightly better than in the absence of lubrication, but lubrication is necessary as successive forming operations increase the risk of titanium buildup on the tool surface and galling. To a greater extent, the influence of lubrication on the depth of the cups is visible for Grade 2 titanium sheet. In the case of both lubricants, i.e., Nos. 2 and 3, the drawn parts were 2.2 mm deeper.

### 3.3. Forming Cylindrical and Hat-Shaped Drawn Parts

**Figure 7:**
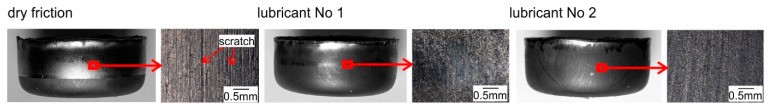
View of formed cylindrical cups.

The lubricant’s effectiveness in forming cylindrically drawn parts was determined on the basis of the value of the maximum forming force as well as on the quality of the outer surface of the cup. A blank holder force of 2.5 kN was used during forming. The results of forming cylindrically drawn parts in conditions of technically dry friction and lubrication with rapeseed oil with the addition of boric acid and boric acid dissolved in methyl alcohol are shown in Figure 7 and Table 5.

Due to the low drawability of the Grade 5 sheet, all drawn parts were damaged—a circumferential crack appeared near the bottom of the drawn part, regardless of whether it was formed in conditions of technically dry friction or in the presence of lubricants.

**Figure 8:**
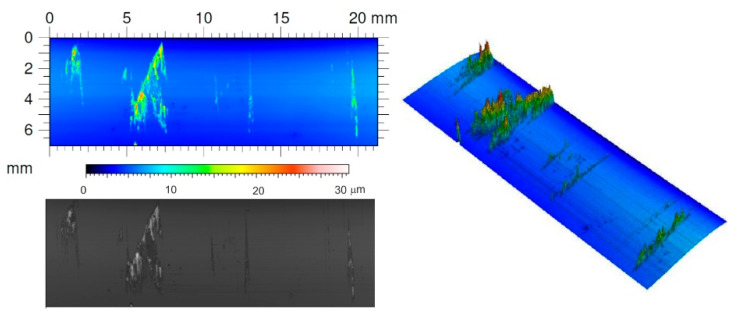
Topography of polished tool surfaces made of NC10 steel after drawing titanium Grade 2 strips.

In the case of forming drawn parts of Grade 2 titanium sheet, the tests showed a positive effect of lubricants with the addition of boric acid on the reduction of the force needed to form the drawn parts compared to forming without lubricant. Slightly better results were obtained for lubricant No. 1, i.e., rapeseed oil with the addition of boric acid. Lubrication resulted in a 38% reduction in drawing force compared to forming in dry conditions for No. 1 lubricant and a 34% reduction for No. 2 lubricant. Additionally, the outer surfaces of the drawn parts were smooth, without scratches, which proved the effective separation of the rubbing surfaces. Breaking the lubricating film always causes the formation of titanium build-up on the working surfaces of tools, which increases their roughness. The presence of build-up on the tool surfaces, especially on the drawing edge of the die, causes the surface of the drawn parts to be scratched. The surface topography of steel tools with visible marks of adhesive wear in the form of titanium Grade2 build-up is shown in Figure 8.

**Figure 9:**
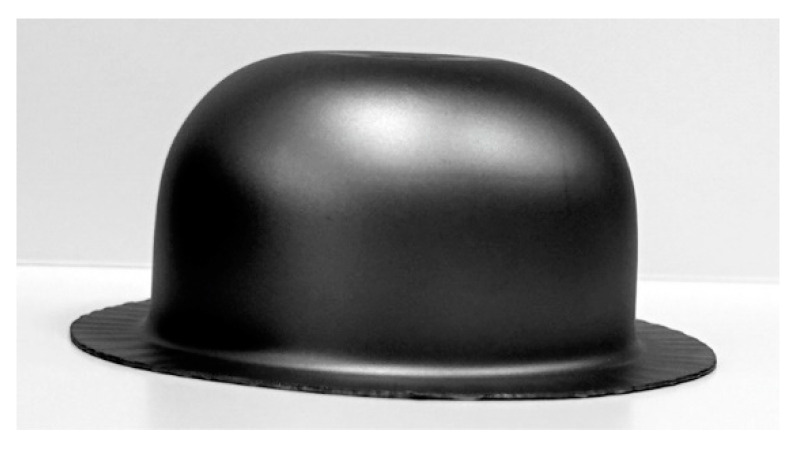
Grade 2 drawn part formed with lubricant No. 2.

Figure 9 shows a hat-shaped part formed in industrial conditions. The part was formed from Grade 2 titanium sheet with lubrication from a 25% solution of boric acid in methyl alcohol (lubricant No. 2). The drawn part has a smooth surface without any signs of scratches.

The use of lubricants with the addition of boric acid during the formation of titanium sheets enables not only the acquisition of drawn parts with good surface quality but also, which is very important from the point of view of environmental protection, the elimination of the previously used mineral oil-based lubricant from the technological process. Mineral oil-based lubricants are difficult to wash off the surface of the drawn parts, and their disposal is expensive. Meanwhile, boric acid is a low-toxic substance. It is only slightly absorbed through intact skin, and because it has antibacterial properties, it is often used in medicine. Boric acid is only poisonous if taken internally or inhaled in large quantities. The proposed lubricants can be effectively washed off the drawn part surfaces: in the case of a 25% solution of boric acid in methyl alcohol with hot water, and in the case of boric acid sprayed on a layer of rapeseed oil with hot water and the addition of a biodegradable detergent, e.g., dishwashing liquid.

### 3.4. FEM Simulation of Forming Hat-Shaped Part

**Figure 10:**
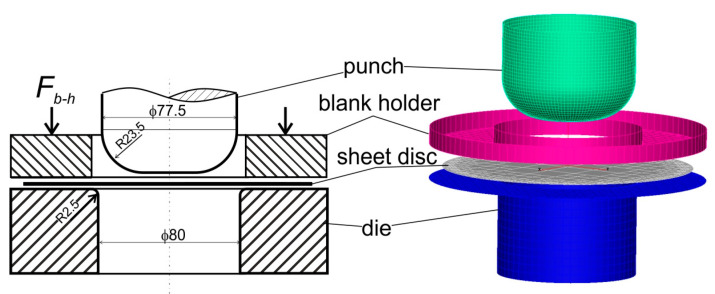
Model of the forming process of the hat-shaped part.

Numerical simulations of the forming process of the hat-shaped parts were carried out using the PAMStamp 2G software based on the finite element method (FEM). This software was specially dedicated to the simulation of sheet metal forming processes. The numerical model of the forming process was developed based on the actual tool and parameters of the forming process (Figure 2b) used in the experiment. The following boundary conditions were assigned to the individual parts of the tool: all degrees of freedom have been removed from the die; the punch and the blank holder can move in the direction of the Z axis; the velocity vector was assigned to the punch; and the force vector *F_b_*_-*h*_ = 12.5 kN was applied to the blank holder (Figure 10). The sheet metal had all degrees of freedom. A mesh of four-node shell elements was generated on the tool and the sheet surfaces.

In the calculations, anisotropic properties of the sheet material were assumed, taking into account the R. Hill plasticity condition [60,61]:

(2) $$\sigma_{p}=\frac{1}{\sqrt{2k}}\sqrt{A{\left(\sigma_{x}-\sigma_{y}\right)}^{2}+B{\left(\sigma_{y}-\sigma_{z}\right)}^{2}+C{\left(\sigma_{z}-\sigma_{x}\right)}^{2}+6\left(D\tau_{xy}^{2}+E\tau_{yz}^{2}+F\tau_{zx}^{2}\right)}$$

where the coefficients *A*, *B*, *C*, *D*, *E*, and *F* are material constants characterizing its anisotropy; *k* is the first-degree function of these coefficients; and *σ_p_* is the yield stress of the isotropic material considered to be equivalent to the anisotropic one.

Assuming that the sheet metal forming process takes place under plane stress conditions, the plasticity condition can be expressed in the form (3):

(3) $$\sigma_{p}=\sqrt{\sigma_{x}^{2}-\frac{2}{1+C}\sigma_{x}\sigma_{y}+\frac{1+B}{1+C}\sigma_{y}^{2}+\frac{6D}{1+C}\tau_{xy}^{2}}$$

In this case, the values of the coefficients *B*, *C*, and *D* can be related to the values of the Lankford normal anisotropy coefficient (4), the value of which varies depending on the angle that forms the axis of the stretched sample with the direction of sheet rolling:

(4) $$r_{\alpha }=\frac{\varphi_{2}}{\varphi_{3}}$$

where α-angle between the direction of sheet rolling and the direction of cutting the sample from the sheet; direction 2 is the direction perpendicular to the rolling direction; and direction 3 is the direction perpendicular to the sheet surface.

Based on the values of the coefficients *r*_0_, *r*_45_, *r*_90_ determined from the tensile test of the samples cut at angles of 0°, 45°, and 90° to the direction of sheet rolling, the sought values of coefficients *B*, *C*, and *D* can be determined (5):

(5) $$B=\frac{1}{r_{90}},\;\;\;\;\;\;\;\;\;C=\frac{1}{r_{0}},\;\;\;\;\;\;\;\;D=\frac{1}{3}\;\left(r_{45}+\frac{1}{2}\right)\left(\frac{1}{r_{0}}+\frac{1}{r_{90}}\right)\;\;$$

The model of the formed material (sheet) was assumed to be elastic-plastic with non-linear reinforcement as described by Equation (6) [60]:

(6) $$\sigma_{p}=C\varepsilon^{n}$$

where *C* is the hardening coefficient and *n* is the hardening exponent.

Basic strength parameters, including normal anisotropy coefficients *r*, hardening coefficient *C,* and hardening exponent *n* of Grade 2 titanium sheet, were determined in a static uniaxial tensile test [59,62].

The properties of the Grade 2 titanium sheet assumed in the calculations are listed in Table 2 and Table 6.

Numerical simulations were carried out for various frictional conditions between the sheet metal and the working surfaces of the tool, taking into account the Coulomb friction model (7):

(7) $$\tau =CoF\cdot \;p_{n}$$

where *τ*—shear stress on the contact surface, *p_n_*—normal pressure.

Two variants of sheet titanium forming were analyzed numerically. In the first variant, the conditions of technically dry friction between the surfaces of the sheet and the working surfaces of the tool were assumed, while in the second variant, lubrication on the contact surface “die–sheet metal–blank holder” and dry conditions on the contact surface “punch–sheet metal” were assumed, corresponding to the conditions in which the experimental tests were performed. The values of the friction coefficient were adopted based on the results of the strip drawing test, i.e., *CoF* = 0.55 in the absence of lubrication and *CoF* = 0.05 for lubricated surfaces (lubricant No. 2).

As a result of the numerical simulations of the sheet metal forming process, distributions of plastic strains in the drawn part and changes in the thickness of its wall were determined.

**Figure 11:**
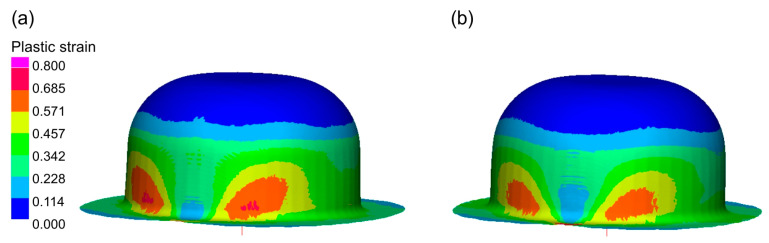
Plastic strain distribution on the drawn part surface: (**a**) dry friction; (**b**) with lubricant.

Figure 11 shows the distribution of plastic strains on the surfaces of the drawn parts.

The results of the numerical simulation show differences in the distribution of plastic strains depending on the assumed frictional conditions. The use of lubrication caused a more even distribution of plastic strains in the material of the drawn part.

**Figure 12:**
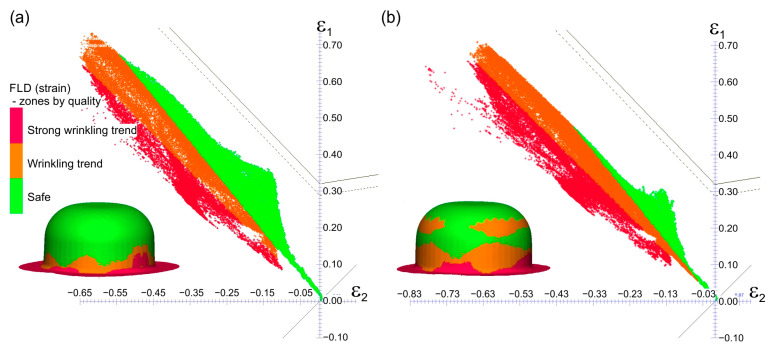
Evaluation of the deep drawing process using the forming limit diagram (zones by quality): (**a**) 1st variant of forming with dry friction; (**b**) 2nd variant of forming with lubrication.

Figure 12 presents the results of strain assessment based on the comparison of the calculated strain values in the cups with the forming limit diagram of the Grade 2 titanium sheet. This FLD was determined experimentally according to the methodology described in [63]. FLDs are a graphical representation of major *ε*_1_ and minor *ε*_2_ limit strains of a given sheet, beyond which local thinning occurs. This may result in cracking of the formed sheet. The PamStamp 2G system also has a “zones by quality” function that indicates not only areas at risk of cracking but also areas with a tendency for the material to wrinkle.

Both in the case of forming without lubrication and in the presence of lubrication, no dangerous areas with a tendency for the material of the drawn part to crack were observed. Lubrication resulted in a decrease in tensile stresses at the expense of an increase in compressive stresses, and in the material of the drawn part, the tendency to wrinkle increased. A similar trend was observed during the experimental deep drawing process.

**Figure 13:**
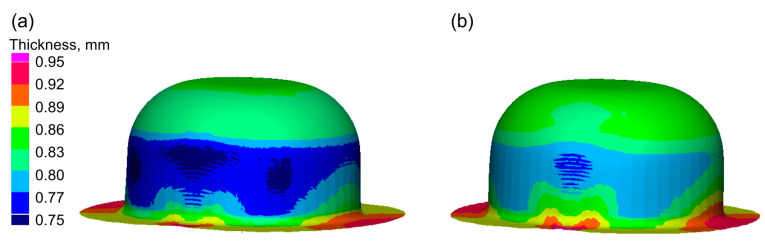
Thickness distribution in the drawn part wall: (**a**) dry friction; (**b**) with lubricant.

In Figure 13, distributions of drawn part wall thickness are presented.

Greater thinning of the drawn part walls is observed in the case of forming under dry friction conditions. Lubrication during forming results in a more even distribution of strains in the cylindrical part of the cup, which reduces the thinning of its wall.

In the flange part, an increase in the thickness of the sheet is observed, resulting from the presence of circumferential compressive stresses.

**Figure 14:**
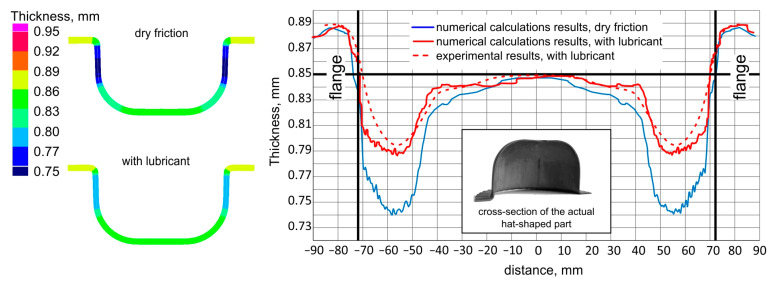
Wall thickness distribution: comparison of experimental and numerical results.

The comparison of the wall thickness on the drawn part section obtained as a result of numerical simulations and experimental tests is shown in Figure 14.

The graph (Figure 14) shows a clear influence of lubrication (continuous red line) on the reduction of wall thinning in comparison to the drawn part formed in the conditions of technically dry friction (continuous blue line). There is also a quite good convergence between the wall thicknesses of the drawn part measured in the experiment (red dashed line) and the thicknesses obtained from numerical calculations (solid red line). Such convergence indicates the possibility of further analyses of the sheet titanium forming process based on numerical calculations.

## 4. Conclusions

On the basis of the carried-out experiments and numerical simulations of the sheet titanium forming process, the following conclusions can be drawn:

- Titanium, especially titanium Grade 2, is extremely susceptible to creating build-up on the forming tools;
- When forming titanium sheets, including Grade 2 and Grade 5 sheets, the role of technological lubricants is not only limited to reducing frictional resistance; above all, they must effectively separate the rubbing surfaces to prevent titanium from sticking to the steel forming tools;
- Lubrication with rapeseed oil alone is not very effective, especially when the normal pressure increases. In the case of polished tools, the use of rapeseed oil has no significant effect on reducing the friction coefficient;
- The addition of boric acid extends the durability of the lubricating film;
- The use of lubricants with the addition of boric acid:
  - Enables more even distribution of plastic strains in the drawn parts—decreases thinning of drawn part wall (Erichsen cupping test, forming of hat-shaped parts, and numerical simulations—Figure 11, Figure 13 and Figure 14);
  - Allows for greater depth of drawn parts—IE increases (Erichsen cupping test—Table 4);
  - Decreases the forming force Ff (forming of cylindrical cups—Table 5);
  - Eliminates the buildup of the deformed materials on the tool surface and thus reduces scratches on the surfaces of the drawn parts;
- In the case of lubrication with boric acid applied to the rapeseed oil layer (lubricant No. 1), the influence of the method of tool surface preparation on the value of frictional resistance was observed—higher values of *CoF* occur in the case of a ground tool—which may indicate the presence of mixed friction (local breakage of the lubricating film);
- Although the strip drawing test shows that boric acid lubricants (No. 1 and No. 2) effectively reduce the coefficient of friction, additional tests such as the Erichsen cupping test, where biaxial tensile predominates, are needed to fully assess the lubricant’s effectiveness;
- Experimental studies have shown that the method of spraying boric acid on a layer of vegetable oil is better than spraying a sheet with a 25% solution of boric acid in methyl alcohol because the layer formed after the evaporation of alcohol is more susceptible to cracking, which leads to direct contact of the deformed sheet with the tool and causes an increase in *CoF*;
- Boric acid as a lubricant is an excellent alternative to graphite and MoS_2_, the main disadvantage of which is the formation of hard-to-remove dirt on the surface of the drawn parts.

## Figures and Tables

**Table 1:** Nominal chemical composition of Grade 2 and Grade 5 titanium.

Material	Element, wt.%							
	Al	V	Fe	O	C	N	H	Ti
Grade 2	-	-	≤0.3	≤0.25	≤0.08	≤0.08	≤0.015	balance
Grade 5	5.5–6.75	3.5–4.5	≤0.4	≤0.2	≤0.08	≤0.05	≤0.015	balance

**Table 2:** Mechanical properties of analyzed sheets.

Material	Offset Yield Point R_p0.2_, MPa	Tensile Strength R_m_, MPa	Elongation A_10_, %
Grade 2	354	472	25
Grade 5	870	1090	11

**Table 3:** Roughness of strip surfaces.

Material	Roughness Parameter	
	Ra, µm	Rz, µm
Grade 2	~0.71	~4.8
Grade 5	~0.61	~4.1

**Table 4:** Results of the Erichsen cupping test.

Material	Erichsen Index *IE*, mm		
	Technically Dry Friction	Lubricant No 1	Lubricant No 2
Grade 2	11.2	13.4	13.4
Grade 5	5.0	6.1	5.4

**Table 5:** Results of cylindrical cups forming.

Material	Maximum Forming Force *F_f_*, kN		
	Technically Dry Friction	Lubricant No 1	Lubricant No 2
Grade 2	35.5	22	23.5
Grade 5	fracture	fracture	fracture

**Table 6:** Material data assumed in numerical calculations.

Lankford coefficient	*r*_0_	2.49
	*r*_45_	4.50
	*r*_90_	5.20
Hardening coefficient *C*, MPa		724.4
Strain hardening exponent *n*		0.144

## Data Availability

Not applicable.
